# Neurological and immunological characteristics of a novel immortalized bovine brainstem-derived cell line and its susceptibility to arbovirus infection

**DOI:** 10.3389/fcimb.2025.1518808

**Published:** 2025-02-13

**Authors:** Rina Ikeda, Tohru Yanase, Misako Konishi, Katsunori Murota, Shogo Tanaka, Takato Takenouchi

**Affiliations:** ^1^ Kagoshima Research Station, National Institute of Animal Health, National Agriculture and Food Research Organization, Kagoshima, Kagoshima, Japan; ^2^ Institute of Agrobiological Sciences, National Agriculture and Food Research Organization, Tsukuba, Ibaraki, Japan

**Keywords:** arbovirus, bovine brainstem cells, immortalization, immunomodulatory molecule, neuronal differentiation

## Abstract

Immortalized bovine neuronal cell lines provide a reliable *in vitro* model for studying interactions with bovine infectious pathogens that target the host nervous system. Although we previously established an immortalized fetal bovine brain-derived FBBC-1 cell line, there are currently no other bovine neuronal cell lines commonly available. In the present study, we developed a novel immortalized cell line, IKBM, derived from the adult bovine brainstem by transferring a SV40 large T antigen gene using lentiviral vectors, and compared its characteristics to the FBBC-1 cell line. As with FBBC-1 cells, IKBM cells extended neurite-like processes in response to agents that increase cytosolic cyclic AMP levels. A comprehensive analysis using RNA sequencing demonstrated that both cell lines potentially possess neural progenitor cell-like properties and differentiate into dopaminergic neuron-like cells after induction of the outgrowth of neurite-like processes. Unexpectedly, we found that the mRNAs of multiple immunomodulatory molecules were highly expressed in IKBM cells, but not in FBBC-1 cells. Although IKBM cells were susceptible to infection with arboviruses (Akabane and Chuzan viruses) that cause neurological symptoms in cattle, viral titers were lower in IKBM cell cultures than in hamster lung-derived HmLu-1 cell cultures, which are frequently used to isolate arboviruses. The reduced production of viruses in IKBM cell cultures may be related to the high expression of immunomodulatory molecules in these cells. Therefore, IKBM and FBBC-1 cell lines offer the opportunity to develop unique *in vitro* models of the bovine nervous system for the study of host-pathogen interactions based on their respective properties.

## Introduction

Outbreaks of infectious diseases in cattle cause severe economic damage to the beef and dairy industries. Infectious pathogens in cattle target not only the respiratory and digestive systems, but also the nervous system. Bovine spongiform encephalopathy (BSE) is a fatal neurodegenerative disease of cattle caused by a proteinaceous pathogen “prion”, which replicates in nerve cells through the conversion of a normally expressed prion protein (PrP^C^) into an abnormal pathogenic isoform (PrP^Sc^) (Orge et al., 2021). Listeriosis is a zoonotic disease caused by the ubiquitous Gram-positive bacterium *Listeria monocytogenes*, some strains of which exhibit neurotropic potential that enhances their intra-axonal spread within nerves (Henke et al., 2015). Several arthropod-borne viruses (arboviruses) are also neurotropic and cause severe neurological disorders in cattle (Yanase et al., 2020; Kitani et al., 2000).

Due to facility and biosafety issues, it is not easy to conduct *in vivo* pathogen infection experiments using large livestock animals, such as cattle. Therefore, *in vitro* cell culture models are beneficial for investigating host-pathogen interactions at the molecular and cellular levels. To develop an *in vitro* model for studying bovine neurotropic pathogens, we previously established the immortalized fetal bovine brain-derived cell line, FBBC-1 (Takenouchi et al., 2009). FBBC-1 cells failed to support the propagation of BSE-derived PrP^Sc^ (Takenouchi et al., 2009), and were found to be susceptible to infection with *L. monocytogenes* (Henke et al., 2015; Gözel et al., 2019) and bovine herpesvirus type-1 (Rudd et al., 2021).

Despite their usefulness as *in vitro* infection models, there are currently no other bovine neuronal cell lines commonly available. In the present study, we established a new immortalized cell line derived from the adult bovine brainstem, the Immortalized Kagoshima bovine brain medullary tissue-derived cell line (IKBM), by introducing the SV40 large T antigen (SV40LT) gene and compared its characteristics with those of the FBBC-1 cell line.

## Materials and methods

### Ethics statement

The protocols for the use of animals were approved by the Animal Ethics Committee of the National Institute of Animal Health (NIAH), National Agriculture and Food Research Organization (NARO) (approval No. R5-R13-NIAH).

### Primary culture of bovine brainstem tissue-derived cells

The brainstem tissue of a 49-month-old Japanese black cow was collected at Kagoshima Research Station, NIAH, NARO, Japan. The medulla oblongata (gray matter) was dissected out, minced using a nylon mesh (square size of 0.512 mm), and digested by an incubation with collagenase-dispase (Roche Diagnostics, Basel, Switzerland)/Dulbecco’s phosphate-buffered saline (DPBS) solution (1 mg/mL) containing DNase I (Roche Diagnostics) (40 μg/mL) at 37°C for 1 h. After filtering the digested tissue through a 100-μm cell strainer (Corning, Glendale, AZ), cells were collected from the eluate by centrifugation (1500 rpm for 5 min) and re-suspended in growth medium composed of Dulbecco’s modified Eagle’s medium (DMEM) (Nacalai Tesque, Inc., Kyoto, Japan) containing 10% heat-inactivated fetal bovine serum (FBS) (FUJIFILM Wako Pure Chemical Corp., Osaka, Japan) supplemented with 25 μM monothioglycerol (FUJIFILM Wako), 10 μg/mL insulin (Sigma), streptomycin-penicillin (100 μg/mL and 100 U/mL, respectively) (Nacalai Tesque), and 5 μg/mL Fungin (InvivoGen, San Diego, CA). The cell suspension was added to T-75 tissue culture flasks (Sumitomo Bakelite Co., Ltd., Tokyo, Japan) and cultured at 37°C in a humidified atmosphere of 95% air/5% CO_2_. The culture medium was replaced every 3-4 days. After 3-4 weeks, primary cultured cells were detached using TrypLE express solution (Thermo Fisher Scientific, Waltham, MA), and replated into 60-mm tissue culture dishes (Corning).

### Establishment and subculturing of IKBM cells

Lentiviral particles carrying the SV40LT and neomycin-resistance genes were prepared as previously described (Takenouchi et al., 2017). Primary cells were infected with lentiviral particles in the presence of 6 μg/mL of Polybrene (Nacalai Tesque) for 24 h. After further culturing for 2 days, the treatment of cells with medium containing 400 μg/ml G418 (Nacalai Tesque) was initiated. G418-resistant cells were expanded and subsequently established as IKBM cells.

Regarding subculturing, IKBM cells were re-suspended in neuronal growth medium composed of DMEM/Ham’s F-12 (DF) (Nacalai Tesque) containing 10% FBS supplemented with 50 ng/ml recombinant human epidermal growth factor (Sigma), 50 ng/ml recombinant human basic fibroblast growth factor (Sigma), streptomycin-penicillin (100 μg/mL and 100 U/mL, respectively), and N-2 Supplement (Thermo Fisher Scientific). Cells (1×10^6^) were seeded on 100-mm tissue culture dishes (TPP Techno Plastic Products AG, Trasadingen, Switzerland) and continuously passaged every 4-5 days. At each passage, cells were detached using TrypLE express solution, and the number of harvested cells was measured using a Bio-Rad TC20 automated cell counter. The subculturing of FBBC-1 cells was also performed according to the same protocol.

### Induction of the outgrowth of neurite-like processes

IKBM cells suspended in neuronal growth medium were treated with 100 μM forskolin (Nacalai Tesque) or 5 mM dibutyryl-cyclic AMP (dbcAMP) (Nacalai Tesque), and were then plated onto 35-mm tissue culture (Corning) or non-tissue culture dishes (Corning). FBBC-1 cells suspended in FBS-reduced neuronal growth medium (0.1% FBS) were simultaneously treated with 100 μM forskolin and 2 mM dbcAMP, and were then plated onto 35-mm tissue culture dishes. Since dimethyl sulfoxide (DMSO) (Nacalai Tesque) was used to dissolve forskolin, DMSO (0.1%) was added to untreated cells as the control. The outgrowth of neurite-like processes was examined under a phase-contrast microscope (Leica, Bensheim, Germany).

### Immunocytochemistry

IKBM cells were seeded on 8-well chamber glass slides (2×10^5^ cells/well) (Asahi Glass Co., Ltd., Tokyo, Japan). The next day, cells were washed with DPBS and fixed using acetone:methanol (1:1, v/v) at –30°C for 30 min. After being washed with phosphate-buffered saline (PBS) containing 0.05% Tween 20 (PBST), cells were permeabilized with 1% Triton X-100/PBS for 10 min and blocked with Blocking One Histo (Nacalai Tesque) for 30 min. Cells were then incubated with a mouse monoclonal antibody against SV40LT (Oncogene Science, Cambridge, MA) at room temperature for 1 h in a humidified box. After rinsing the slides with PBST, the EnVision system (DAKO, Hamburg, Germany) was used to visualize antibody-antigen reactions according to the manufacturer’s procedure. Cell nuclei were counterstained with Mayer’s hematoxylin solution (FUJIFILM Wako), and stained slides were examined using a microscope.

### RNA sequencing analysis

IKBM cells (5×10^5^) were cultured in 35-mm tissue culture dishes in the absence or presence of 100 μM forskolin for 24 h. FBBC-1 cells were also cultured in 35-mm tissue culture dishes in the absence or presence of both 100 μM forskolin and 2 mM dbcAMP for 24 h. Total RNA was then extracted from cells using the Cytiva RNAspin mini isolation kit (Thermo Fisher Scientific). RNA sequencing (RNA-seq) analyses were performed at Seibutsugiken Co., Ltd. (Kanagawa, Japan). Three independent experiments were performed and the differential expression of genes was analyzed using the iDEP.96 website (http://bioinformatics.sdstate.edu/idep96/). Transcripts per million (TPM) data are expressed as the mean ± standard error of the mean (SEM), and mean values were analyzed with an unpaired *t*-test using the software GraphPad InStat 3 for Windows. The significance of differences was set at p <0.05.

RNA-seq data were also utilized to investigate contamination by microorganisms in IKBM and FBBC-1 cell cultures. Briefly, the FASTQ-formatted files obtained from the RNA-seq analysis were initially mapped to the entire *Bos taurus* genome sequence as a reference. The resulting unmapped reads were then *de novo* assembled into contiguous sequences, and the generated contigs were subsequently analyzed using BLAST. All data processes were performed using CLC genomic workbench 23 (QIAGEN, Hilden, Germany).

### Arbovirus growth assay

IKBM and hamster lung (HmLu-1) cells were seeded at 2×10^5^/well in 24-well tissue culture plates (Sumitomo Bakelite). The next day, cells were inoculated with arboviruses, such as the Akabane virus (AKAV) OBE-1 strain and Aino virus (AINOV) JaNAr28 strain, at a multiplicity of infection (MOI) of 0.01. Cells were also inoculated with Chuzan virus (CHUV) isolate 31 and the D'Aguilar virus (DAGV) KSB-29/E/01 strain at a MOI of 0.1. MOI was calculated with the 50% tissue culture infectious dose (TCID_50_) values of each virus stock. After cells had been incubated at 37°C for 1 h, the inoculum was removed and cells were washed three times with DPBS. DF medium containing 2% FBS and Eagle’s minimum essential medium (Shimazu, Kyoto, Japan) containing 0.3% tryptose phosphate broth (Becton Dickinson and Company, Franklin Lakes, NJ) were added to IKBM and HmLu-1 cells, respectively. Culture supernatants were collected 0, 12, 24, 48, 72, 96, and 120 hours post-infection (hpi). Virus titrations were analyzed using cytopathic effects (CPEs) against HmLu-1 cells. Briefly, HmLu-1 cells suspended in 100 µL of ten-fold serially diluted supernatant samples were seeded on 96-well cell culture plates (2.5×10^4^/well) (Sumitomo Bakelite). After a 7-day incubation, the presence of CPEs was assessed by the disruption of monolayer HmLu-1 cell sheets caused by viral infection under a microscope. Viral titers were expressed as TCID_50_/mL. The viral growth assay was independently performed three times for each arbovirus, and data are expressed as the mean ± standard deviation (SD). Mean values were analyzed with a one-way analysis of variance followed by Dunnett’s *post-hoc* test using the software GraphPad InStat 3 for Windows. The significance of differences was set at p <0.05.

## Results

### Establishment of IKBM cells

**Figure 1:**
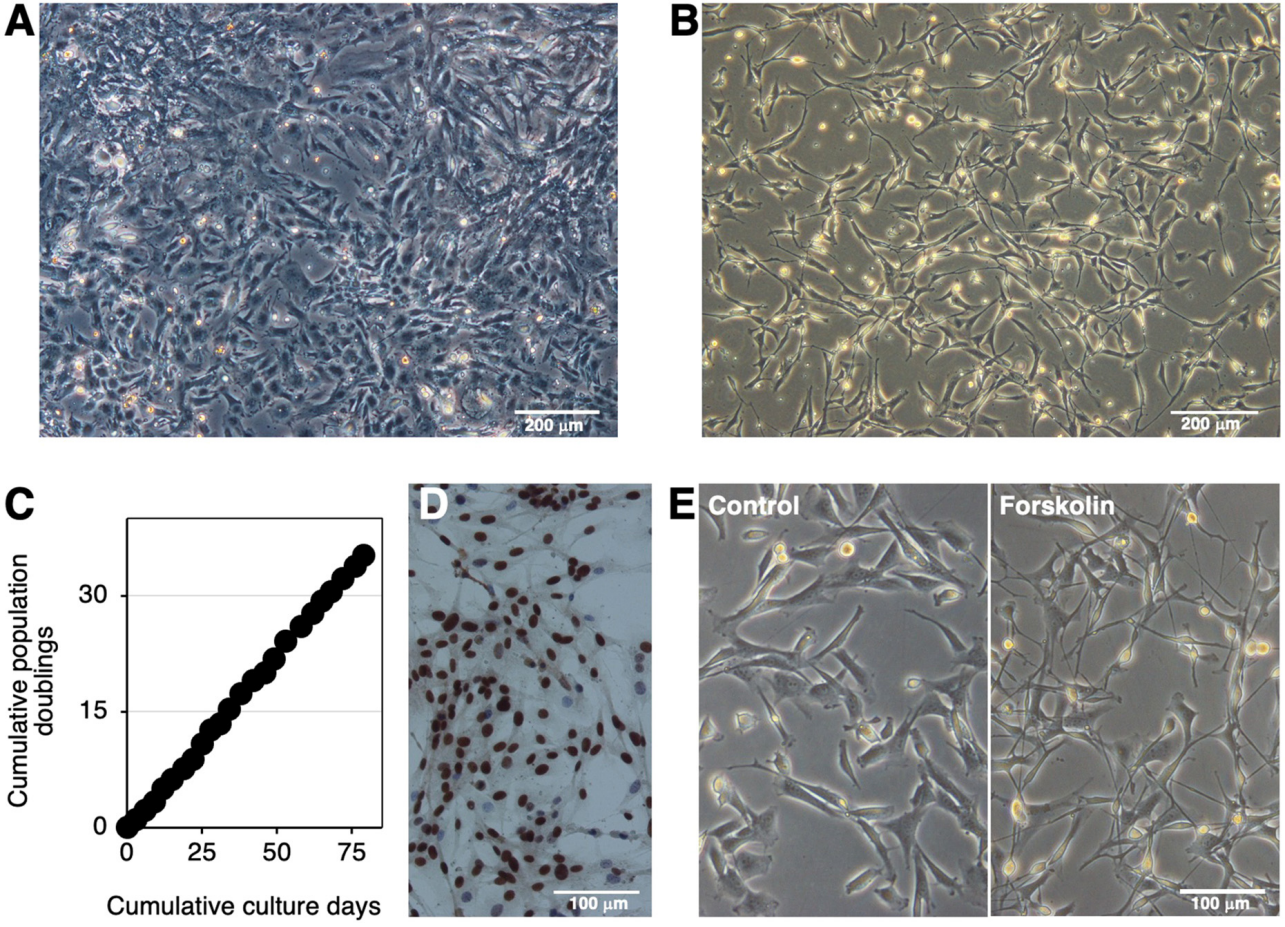
Establishment of the IKBM cell line. The morphology of the bovine brainstem medullary tissue-derived primary cells **(A)** or IKBM cells **(B)** established was examined under a phase-contrast microscope. The cumulative population doublings of IKBM cells were plotted against the duration of the culture period (in days) **(C)**. SV40LT protein expression was detected in the nuclei of IKBM cells [*brown*, **(D)**]. All nuclei were counterstained with hematoxylin [*blue*, **(D)**]. IKBM cells were cultured in the absence (*Control*) or presence of 100 μM forskolin (*Forskolin*) in 35-mm tissue culture dishes for 8 h **(E)**. Images are representative of at least two independent experiments.

Cells derived from bovine brainstem medullary tissue were primary cultured in tissue culture dishes (**Figure 1A**). The SV40LT gene was then introduced into the primary cells using a lentiviral vector. After selection by the G418 treatment, proliferating cells resistant to this treatment were established as IKBM cells, most of which exhibited a spindle-like morphology (**Figure 1B**). IKBM cells proliferated at a doubling time of approximately 2 days and were stably passaged for more than 35 population doublings (**Figure 1C**). Immunocytochemistry revealed SV40LT protein expression in the nuclei of IKBM cells (**Figure 1D**).

### Induction of the outgrowth of neurite-like processes in IKBM cells

Agents that increase cytosolic cAMP levels, such as forskolin and dbcAMP, are known as inducers of neuronal differentiation. The treatment with forskolin extended neurite-like processes in IKBM cells (**Figure 1E**). Upon the forskolin treatment, morphological changes in IKBM cells occurred immediately within 4 h, and the outgrowth of neurite-like processes reached almost the maximum after 1 day (**Supplementary Figure S1**). The treatment with dbcAMP also began to induce the outgrowth of neurite-like processes after 1 day of treatment (**Supplementary Figure S1**).

We attempted to establish optimal treatment conditions for the outgrowth of neurite-like processes in FBBC-1 cells. The results obtained revealed that the simultaneous treatment with forskolin and dbcAMP exerted synergistic effects on the outgrowth of neurite-like processes in FBBC-1 cells (**Supplementary Figure S2**). On the other hand, no visible synergistic effects on the outgrowth of neurite-like processes were observed in IKBM cells upon the combined treatment with both agents.

### Neural progenitor cell-like characteristics of IKBM and FBBC-1 cells

A comprehensive analysis of mRNA expression in IKBM and FBBC-1 cells was performed using RNA-seq. We initially examined the mRNA expression levels of 11 housekeeping genes (Vandesompele et al., 2002; Kuchipudi et al., 2012) in both cell lines. The expression levels of the 18S ribosomal RNA (*RN18S1*), ribosomal protein L13a (*RPL13A*), tyrosine 3-monooxygenase/tryptophan 5-monooxygenase activation protein zeta (*YWHAZ*), β2-microglobulin (*B2M*), hydroxymethylbilane synthase (*HMBS*), and TATA-box binding protein (*TBP*) genes did not significantly change in both cell lines treated and untreated with cAMP-elevating agents (**Supplementary Figure S3**), suggesting the reliability of these reference genes.

**Figure 2:**
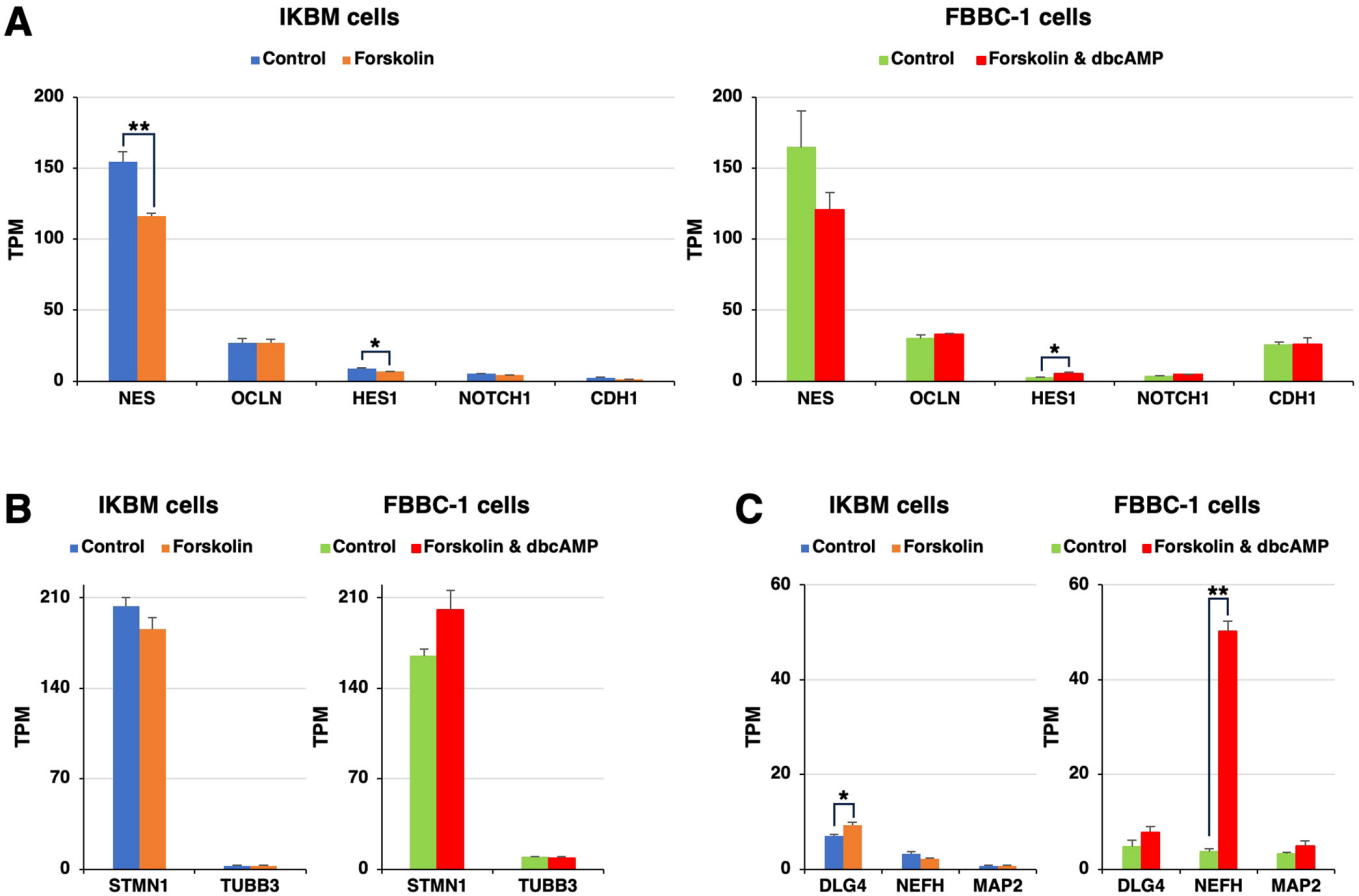
The mRNA expression of neural markers in IKBM and FBBC-1 cells. Total RNA was recovered from IKBM cells untreated (*blue bars*) and treated (*orange bars*) with 100 μM forskolin for 24 h. Total RNA was also recovered from FBBC-1 cells untreated (*green bars*) and treated (*red bars*) with both 100 μM forskolin and 2 mM dbcAMP for 24 h. RNA-seq experiments were performed independently three times. The TPM values of the neuroepithelial cell marker **(A)**, immature neuron marker **(B)**, and mature neuron marker **(C)** genes indicated are expressed as mean ± SEM values (**p <0.01, *p <0.05 vs. Untreated control).

Using the same RNA-seq data, the expression of neuroepithelial cell marker mRNAs, such as nestin (*NES*), occludin (*OCLN*), hairy and enhancer of split-1 (*HES1*), neurogenic locus notch homolog protein 1 (*NOTCH1*), and epithelial cadherin (*CDH1*), was detected in both cell lines, regardless of the treatment with cAMP-elevating agents (**Figure 2A**). Regarding immature neuron markers, stathmin 1 (*STMN1*) and βIII-tubulin (*TUBB3*) mRNAs were expressed to a similar extent in both cell lines (**Figure 2B**). Mature neuron marker mRNAs, such as discs large homolog 4 (*DLG4*), neurofilament heavy (*NEFH*), and microtubule-associated protein 2 (*MAP2*), were constitutively expressed in both cell lines (**Figure 2C**). The mRNA expression level of *NEFH* was 13-fold higher in FBBC-1 cells treated with cAMP-elevating agents than in untreated control cells (**Figure 2C**).

### Dopaminergic neuron-like properties of IKBM and FBBC-1 cells treated with cAMP-elevating agents

To identify the neuronal cell types of IKBM and FBBC-1 cells, we examined the mRNA expression of neuronal cell type-specific genes. We found that the mRNA expression level of nuclear receptor subfamily 4 group A member 2 (*NR4A2*), a known marker of dopaminergic neurons (Sacchetti et al., 2006), was significantly increased in IKBM and FBBC-1 cells after the treatment with cAMP-elevating agents (**Supplementary Figure S4A**). The mRNA expression of tyrosine hydroxylase (*TH*), another dopaminergic neuron marker, slightly increased in FBBC-1 cells after the treatment with cAMP-elevating agents (**Supplementary Figure S4A**).

We then examined the mRNA expression of the genes related to dopamine production. The mRNA expression levels of D-amino acid oxidase (*DAO*) and aromatic L-amino acid decarboxylase (*DDC*) were significantly higher in IKBM cells treated with cAMP-elevating agents than in untreated control cells (**Supplementary Figure S4B**). GTP cyclohydrolase 1 (*GCH1*) and *DDC* mRNA expression levels were also up-regulated in FBBC-1 cells treated with cAMP-elevating agents (**Supplementary Figure S4B**).

### Expression of immunomodulatory molecules in IKBM cells

**Figure 3:**
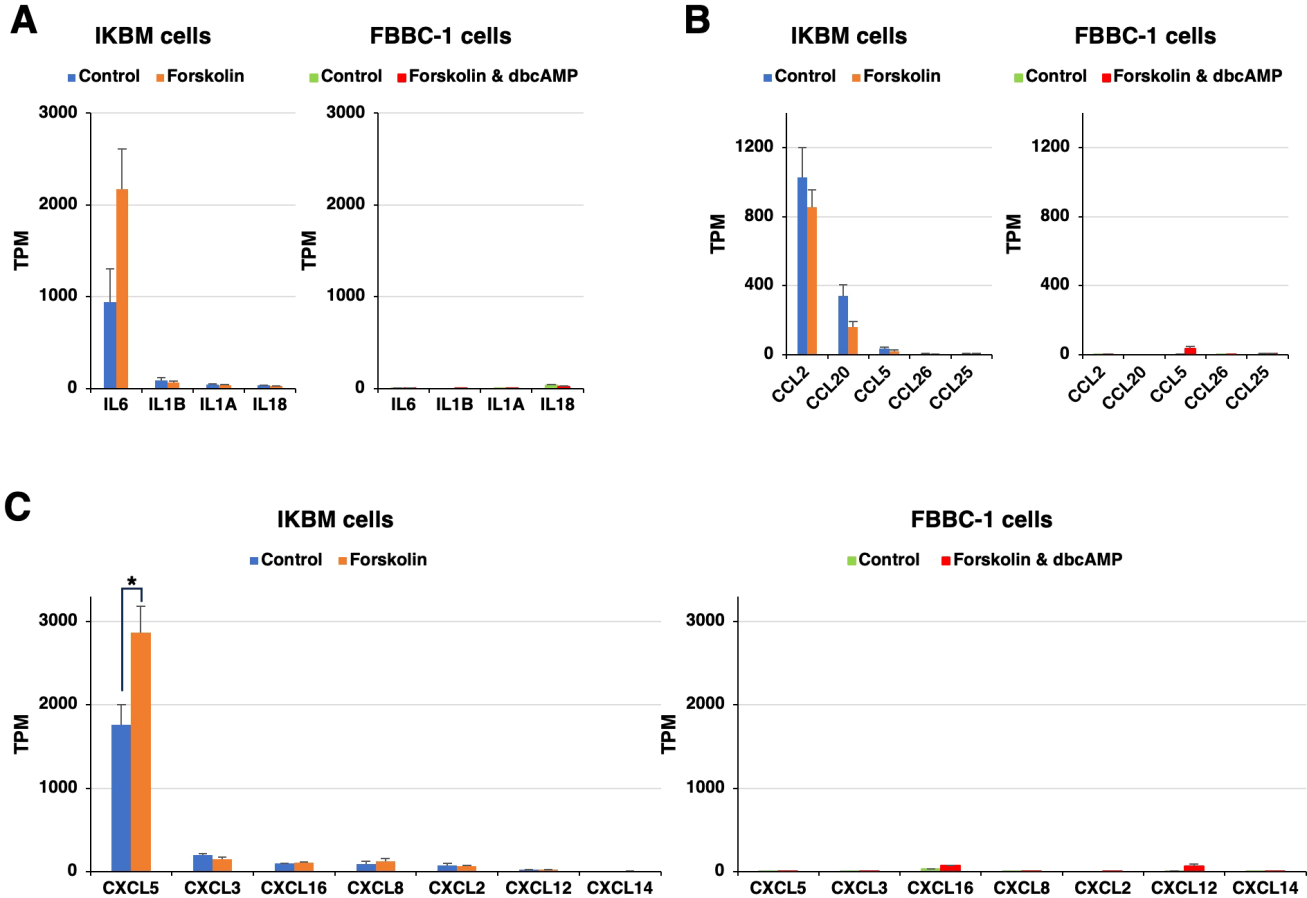
The mRNA expression of immunomodulatory molecules in IKBM and FBBC-1 cells. Total RNA was recovered from IKBM cells untreated (*blue bars*) and treated (*orange bars*) with 100 μM forskolin for 24 h. Total RNA was also recovered from FBBC-1 cells untreated (*green bars*) and treated (*red bars*) with both 100 μM forskolin and 2 mM dbcAMP for 24 h. RNA-seq experiments were performed independently three times. The TPM values of the interleukin (IL) **(A)**, C-C motif chemokine (CCL) **(B)**, and C-X-C motif chemokine (CXCL) **(C)** genes indicated are expressed as mean ± SEM values (*p <0.05 vs. Untreated control).

Previous studies showed that neural progenitor cells play an immunomodulatory role in the central nervous system (Ni et al., 2023). In the present study, we noted that the mRNAs of multiple immunomodulatory molecules were clearly expressed in IKBM cells regardless of the treatment with cAMP-elevating agents (**Figures 3A–C**). The mRNA expression of interleukin-6 (*IL6*) (**Figure 3A**), C-C motif chemokine 2 (*CCL2*) (**Figure 3B**), and C-X-C motif chemokine 5 (*CXCL*5) (**Figure 3C**) was higher in IKBM cells than in FBBC-1 cells.

### Susceptibility of IKBM cells to arbovirus infection

**Figure 4:**
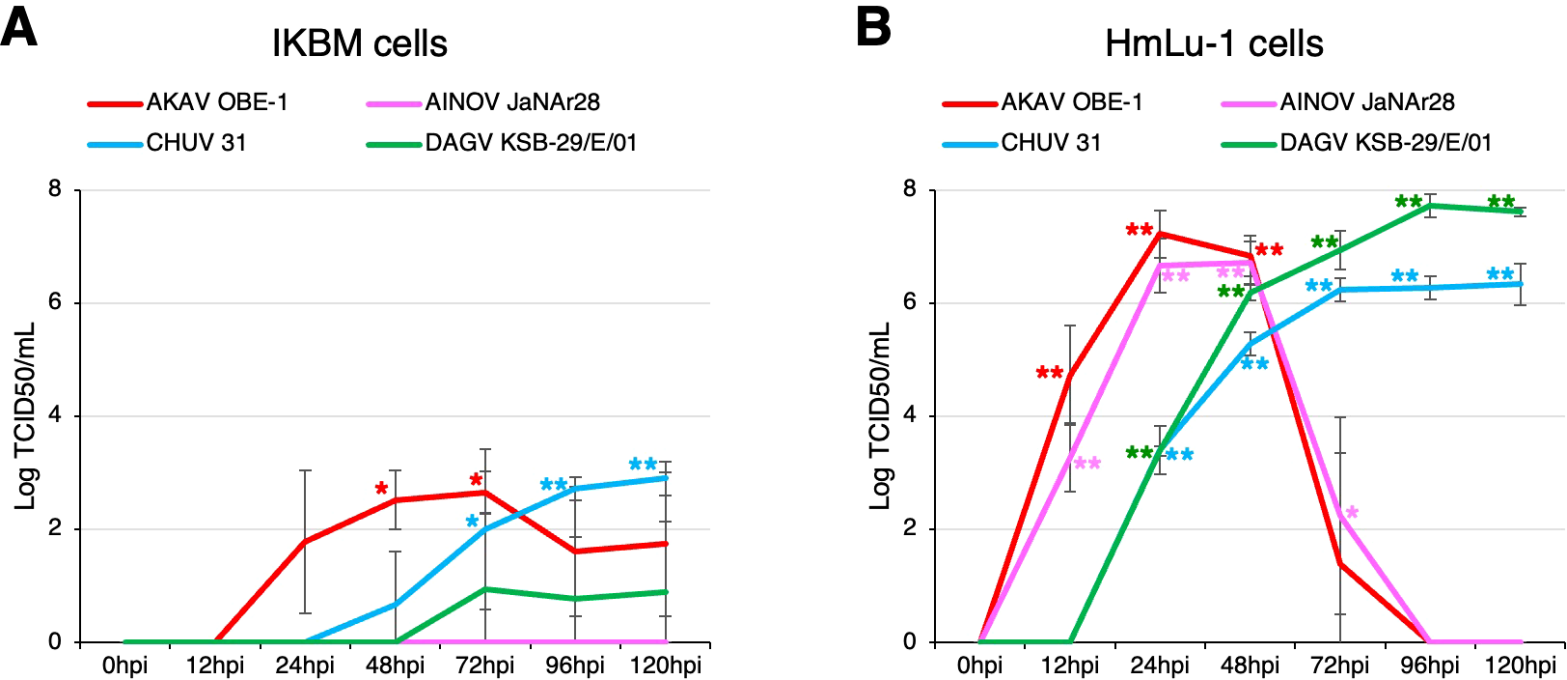
Comparison of arbovirus production in IKBM and HmLu-1 cell cultures. Cell cultures were infected with the AKAV OBE-1 strain (*red line*) and AINOV JaNAr28 strain (*pink line*) (MOI = 0.01). They were also infected with CHUV isolate 31 (*light blue line*) and the DAGV KSB-29/E/01 strain (*green line*) (MOI = 0.1). Culture supernatant samples were collected at the indicated time points post-infection. Viral production in the IKBM **(A)** and HmLu-1 **(B)** cell cultures was estimated by titration experiments with HmLu-1 cells. Data represent the mean ± SD values of three independent experiments (**p <0.01, *p <0.05 vs. 0 hpi). .

We also investigated whether IKBM cells were susceptible to infection with arboviruses that cause neurological symptoms in cattle, and if they support the intracellular replication of these viruses. Although three arboviruses, the AKAV OBE-1 strain, CHUV isolate 31, and DAGV KSB-29/E/01 strain, propagated in IKBM cell cultures (**Figure 4A**), their viral titers were lower than those produced in HmLu-1 cell cultures (**Figure 4B**). The AINOV JaNAr28 strain did not propagate in IKBM cell cultures (**Figure 4A**), but was produced in HmLu-1 cell cultures (**Figure 4B**). Since HmLu-1 cells were highly sensitive to infection with the AKAV OBE-1 and AINOV JaNAr28 strains, their viral titers decreased after 72 hpi due to the disruption of HmLu-1 cell sheets (**Figure 4B**).

## Discussion

Neuronal cell lines from different animal species are commercially available. The rat adrenal gland pheochromocytoma PC12 cell line is one of the most frequently used models for neuroscience research, including studies on neurotoxicity, neuroprotection, neurosecretion, and synaptogenesis (Wiatrak et al., 2020). PC12 cells may be induced to differentiate towards a sympathetic-like neuronal phenotype characterized by the outgrowth of neurite-like processes, in response to treatment with nerve growth factor or cAMP-elevating agents (Hansen et al., 2003; Takenouchi et al., 1999). Mouse neuroblastoma Neuro 2A (N2a) cells, which are derived from the neural crest, also differentiate into dopaminergic neuron-like cells when treated with dbcAMP (Tremblay et al., 2010). Moreover, the dbcAMP treatment induces the neuronal differentiation of human neuroblastoma SH-SY5Y cells towards a noradrenergic phenotype (Kume et al., 2008). Regarding other animal species, we were the first to report the establishment of FBBC-1 cells from fetal bovine brain tissues, in which cAMP-elevating agents induced the outgrowth of neurite-like processes (Takenouchi et al., 2009). Additionally, in the present study, we successfully established another cell line named IKBM derived from adult bovine brainstem tissues. To the best of our knowledge, these are the only two cell lines that may be used as neuronal models in cattle.

The RNA-seq analysis revealed that IKBM and FBBC-1 cells both potentially possess neural progenitor cell-like properties. This is closely relevant to the results showing that the treatment with cAMP-elevating agents induced marked morphological changes with the outgrowth of neurite-like processes, adding dopaminergic neuron-like properties to both cell lines. The significant increase in *NEFH* mRNA expression levels in FBBC-1 cells indicates that these cells were differentiating more efficiently into mature neuronal cells under the treatment conditions presented. Further studies are needed to establish treatment conditions that further enhance the expression levels of mature neuronal markers in IKBM cells.

It is important to note that RNA-seq data showed the high mRNA expression of *IL6*, *CCL2*, and *CXCL*5 in IKBM cells, but not in FBBC-1 cells. Based on the RNA-seq analysis, we also found no evidence of microbial contamination in IKBM and FBBC-1 cell cultures (data not shown). These results suggest that the expression of immunomodulatory molecules was not due to an innate immune response of IKBM cells to foreign substances. The endogenous expression of *IL6* and *CCL2* in IKBM cells may be supported by previous findings showing that *IL6* and *CCL2* mRNAs were expressed in neurons as well as inflammatory cells in the brain (Ringheim et al., 1995; Howe et al., 2017). IKBM cells may be used to investigate the role of these immunomodulatory molecules in host-pathogen interactions in bovine neuronal cells.

Hamster-derived HmLu-1 is one of the cell lines frequently used to isolate arboviruses affecting cattle (Kurogi et al., 1976). All arboviruses tested in the present study efficiently propagated in HmLu-1 cell cultures, suggesting that they had been well adapted to these cells during the isolation process from field-infected bovine samples. Among them, the AKAV OBE-1 strain and CHUV isolate 31 propagated in IKBM cell cultures, whereas the AINOV JaNAr28 strain did not. Although AKAV and AINOV belong to the same Simbu serogroup (Saeed et al., 2001) and cause similar symptoms in ruminants (Yanase et al., 2020), differences in growth efficiency were observed between the virus strains. Therefore, it is likely that the IKBM cell culture model is used to elucidate the molecular mechanisms underlying the differential growth efficiency of these viruses. These findings may be related to the high expression levels of immunomodulatory molecules in IKBM cells, and, thus, additional experiments are required to clarify this possibility.

BSE caused by the proteinaceous pathogen PrP^Sc^ is a well-known zoonotic disease (Orge et al., 2021). The development of an *in vitro* cell culture model that persistently propagates PrP^Sc^ will provide a valuable tool for prion research. Although N2a cells are frequently used to construct the sustained infection model of mouse-adapted PrP^Sc^ (Iwamaru et al., 2012; Takenouchi et al., 2012), BSE-derived PrP^Sc^ is unable to propagate in N2a cell cultures due to the challenges associated with the species barrier. To directly propagate BSE-derived PrP^Sc^, bovine neuronal cell lines expressing endogenous PrP^C^ are considered to be necessary in order to circumvent the species barrier. We previously attempted to persistently propagate BSE-derived PrP^Sc^ using FBBC-1 cells, but have not yet been successful (Takenouchi et al., 2009). Given the mRNA expression of PrP^C^ in IKBM cells (**Supplementary Figure S4C**), this cell line is expected to be a valuable candidate for developing a persistent infection model of BSE-derived PrP^Sc^.

In conclusion, the IKBM cell line described herein has emerged as a useful alternative tool to investigate the interactions between bovine neurotropic pathogens and host brain cells. The IKBM or FBBC-1 cell line offers the opportunity to develop unique *in vitro* models of the bovine nervous system based on their respective properties.

## Data Availability

The original contributions presented in the study are publicly available. This data can be found here: [DDBJ Center (https://ddbj.nig.ac.jp/search)/accession numbers PRJDB20208 and PRJDB20222].

## Supplementary material

Supplementary Figure 1 Induction of the outgrowth of neurite-like processes in forskolin- or dbcAMP-treated IKBM cells. IKBM cells were cultured in the absence (*upper panels*) or presence of 100 μM forskolin (*middle panels*) or 5 mM dbcAMP (*lower panels*) in 35-mm non-tissue culture dishes. The morphology of cells was examined under a phase-contrast microscope at the indicated time points. DMSO (0.1%) was added to untreated cells as the control (*upper panels*). Images are representative of three independent experiments.

Supplementary Figure 2 Induction of the outgrowth of neurite-like processes in FBBC-1 cells by the combined treatment with forskolin and dbcAMP. FBBC-1 cells were cultured in the absence **(A)** or presence of both 100 μM forskolin and 2 mM dbcAMP **(B)** in 35-mm tissue culture dishes for 24 h. The morphology of cells was examined under a phase-contrast microscope. Images are representative of three independent experiments.

Supplementary Figure 3 The mRNA expression of housekeeping genes in IKBM and FBBC-1 cells. Total RNA was recovered from IKBM cells untreated (*blue bars*) and treated (*orange bars*) with 100 μM forskolin for 24 h. Total RNA was also recovered from FBBC-1 cells untreated (*green bars*) and treated (*red bars*) with both 100 μM forskolin and 2 mM dbcAMP for 24 h. RNA-seq experiments were performed independently three times. The TPM values of the higher expression [glyceraldehyde-3-phosphate dehydrogenase (*GAPDH*), β-actin (*ACTB*), *RN18S1*, *RPL13A*, and *YWHAZ*] **(A)** and lower expression [succinate dehydrogenase complex flavoprotein subunit A (*SDHA*), *B2M*, hypoxanthine phosphoribosyltransferase 1 (*HPRT1*), ubiquitin C (*UBC*), *HMBS*, and *TBP*] **(B)** housekeeping genes are expressed as mean ± SEM values (**p <0.01 vs. Untreated control).

Supplementary Figure 4 The mRNA expression of dopaminergic neuron marker and PrP^C^ genes in IKBM and FBBC-1 cells. Total RNA was recovered from IKBM cells untreated (*blue bars*) and treated (*orange bars*) with 100 μM forskolin for 24 h. Total RNA was also recovered from FBBC-1 cells untreated (*green bars*) and treated (*red bars*) with both 100 μM forskolin and 2 mM dbcAMP for 24 h. RNA-seq experiments were performed independently three times. The TPM values of the dopaminergic neuron marker **(A)**, dopamine production-related **(B)**, and PrP^C^ (*PRNP*) **(C)** genes indicated are expressed as mean ± SEM values (**p <0.01, *p <0.05 vs. Untreated control).
