# Supplementary figures and images for: Neurological and immunological characteristics of a novel immortalized bovine brainstem-derived cell line and its susceptibility to arbovirus infection
Source: Front Cell Infect Microbiol. 2025 Feb 13;15:1518808. doi: 10.3389/fcimb.2025.1518808 (PMC11865082; doi:10.3389/fcimb.2025.1518808)

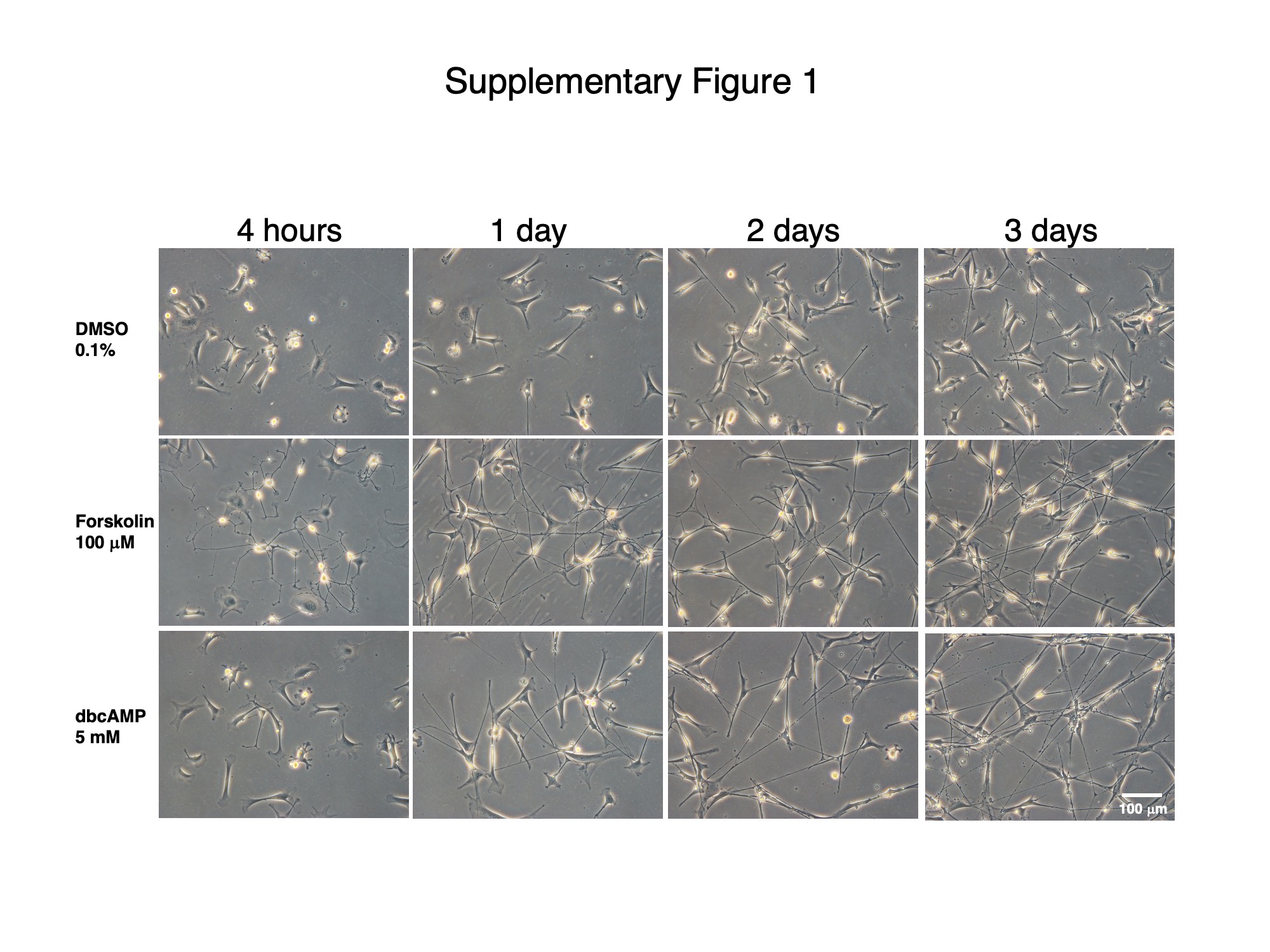

Supplement: Supplementary Figure 1 — Induction of the outgrowth of neurite-like processes in forskolin- or dbcAMP-treated IKBM cells. IKBM cells were cultured in the absence (upper panels) or presence of 100 μM forskolin (middle panels) or 5 mM dbcAMP (lower panels) in 35-mm non-tissue culture dishes. The morphology of cells was examined under a phase-contrast microscope at the indicated time points. DMSO (0.1%) was added to untreated cells as the control (upper panels). Images are representative of three independent experiments. [file Image1.jpeg]

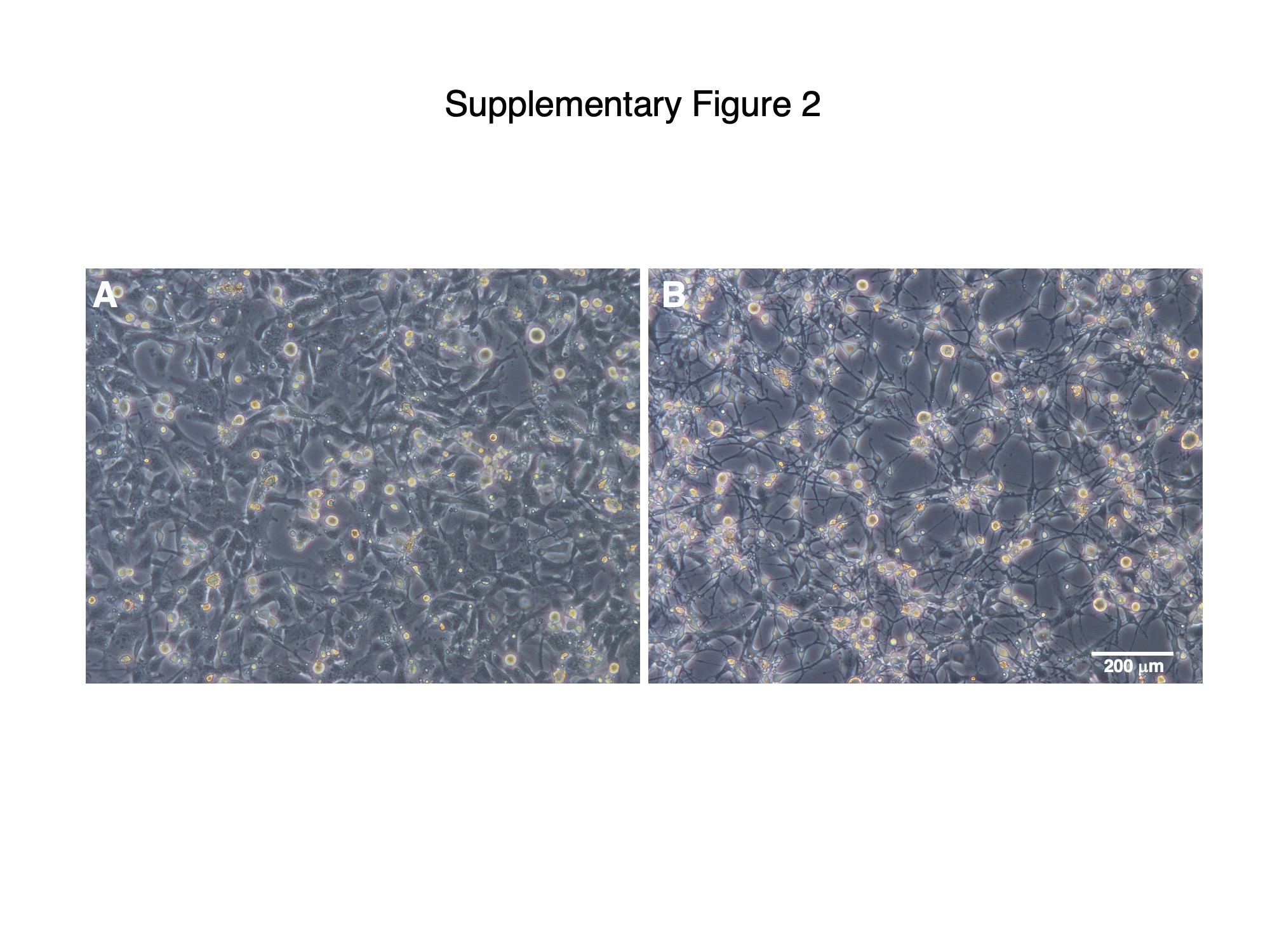

Supplement: Supplementary Figure 2 — Induction of the outgrowth of neurite-like processes in FBBC-1 cells by the combined treatment with forskolin and dbcAMP. FBBC-1 cells were cultured in the absence (A) or presence of both 100 μM forskolin and 2 mM dbcAMP (B) in 35-mm tissue culture dishes for 24 h. The morphology of cells was examined under a phase-contrast microscope. Images are representative of three independent experiments. [file Image2.jpeg]

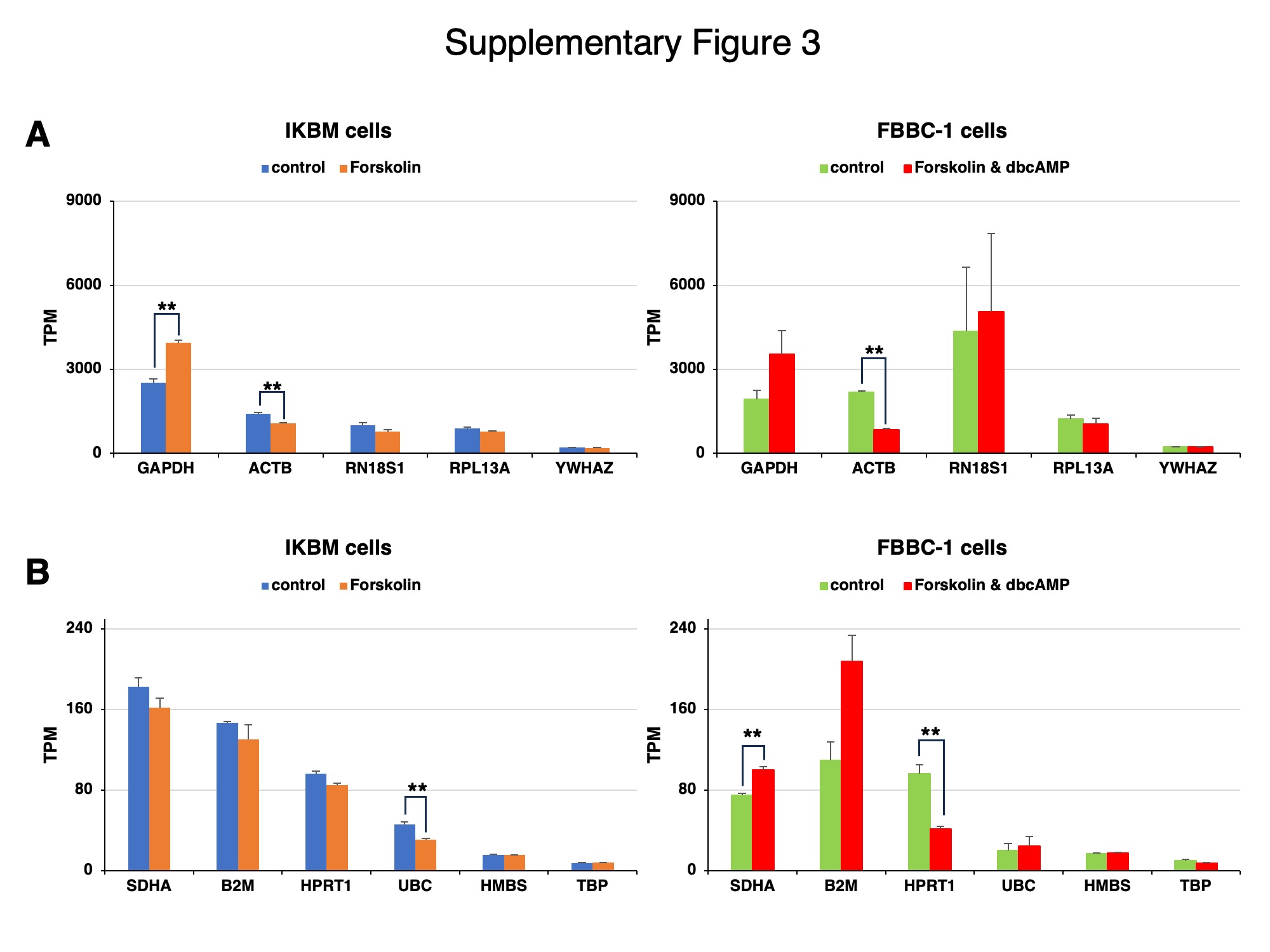

Supplement: Supplementary Figure 3 — The mRNA expression of housekeeping genes in IKBM and FBBC-1 cells. Total RNA was recovered from IKBM cells untreated (blue bars) and treated (orange bars) with 100 μM forskolin for 24 h. Total RNA was also recovered from FBBC-1 cells untreated (green bars) and treated (red bars) with both 100 μM forskolin and 2 mM dbcAMP for 24 h. RNA-seq experiments were performed independently three times. The TPM values of the higher expression [glyceraldehyde-3-phosphate dehydrogenase (GAPDH), β-actin (ACTB), RN18S1, RPL13A, and YWHAZ] (A) and lower expression [succinate dehydrogenase complex flavoprotein subunit A (SDHA), B2M, hypoxanthine phosphoribosyltransferase 1 (HPRT1), ubiquitin C (UBC), HMBS, and TBP] (B) housekeeping genes are expressed as mean ± SEM values (**p <0.01 vs. Untreated control). [file Image3.jpeg]

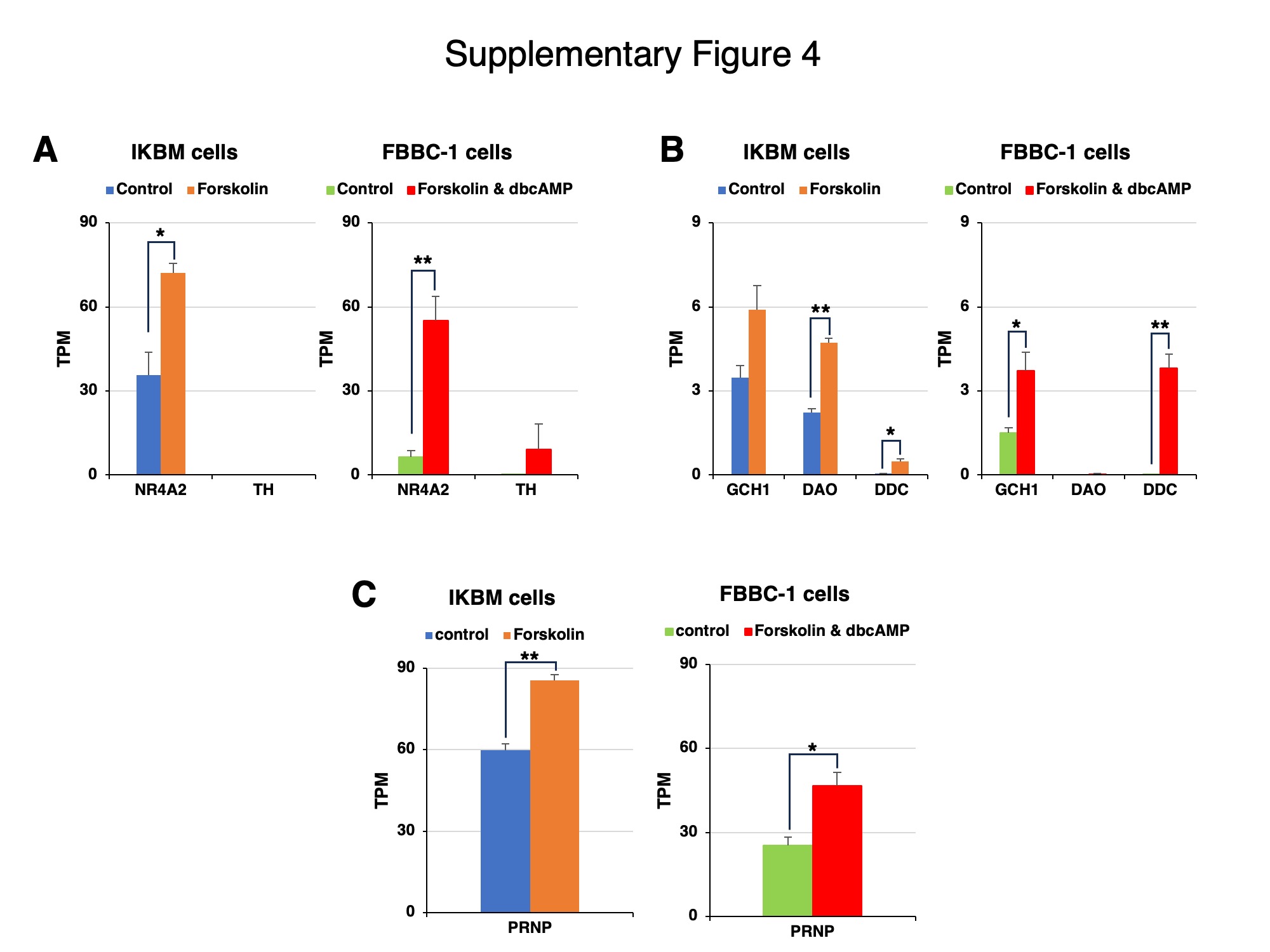

Supplement: Supplementary Figure 4 — The mRNA expression of dopaminergic neuron marker and PrPC genes in IKBM and FBBC-1 cells. Total RNA was recovered from IKBM cells untreated (blue bars) and treated (orange bars) with 100 μM forskolin for 24 h. Total RNA was also recovered from FBBC-1 cells untreated (green bars) and treated (red bars) with both 100 μM forskolin and 2 mM dbcAMP for 24 h. RNA-seq experiments were performed independently three times. The TPM values of the dopaminergic neuron marker (A), dopamine production-related (B), and PrPC (PRNP) (C) genes indicated are expressed as mean ± SEM values (**p <0.01, *p <0.05 vs. Untreated control). [file Image4.jpeg]
